# New genus-specific primers for PCR identification of *Rubrobacter* strains

**DOI:** 10.1007/s10482-019-01314-3

**Published:** 2019-08-12

**Authors:** Jean Franco Castro, Imen Nouioui, Juan A. Asenjo, Barbara Andrews, Alan T. Bull, Michael Goodfellow

**Affiliations:** 1 School of Natural and Environmental Sciences, Ridley Building, Newcastle University, Newcastle upon Tyne, NE1 7RU UK; 2 Department of Chemical Engineering, Biotechnology and Materials, Centre for Biotechnology and Bioengineering (CeBiB), University of Chile, Beauchef 851, Santiago, Chile; 3 School of Biosciences, University of Kent, Canterbury, Kent, CT2 1NJ UK

**Keywords:** Actinobacteria, *Rubrobacter*, Atacama desert, Taxonomy, Genus-specific primers

## Abstract

A set of oligonucleotide primers, Rubro223f and Rubro454r, were found to amplify a 267 nucleotide sequence of 16S rRNA genes of *Rubrobacter* type strains. The primers distinguished members of this genus from other deeply-rooted actinobacterial lineages corresponding to the genera *Conexibacter*, *Gaiella*, *Parviterribacter*, *Patulibacter*, *Solirubrobacter* and *Thermoleophilum* of the class *Thermoleophilia*. Amplification of DNA bands of about 267 nucleotides were generated from environmental DNA extracted from soil samples taken from two locations in the Atacama Desert. Sequencing of a DNA library prepared from the bands showed that all of the clones fell within the evolutionary radiation occupied by the genus *Rubrobacter*. Most of the clones were assigned to two lineages that were well separated from phyletic lines composed of *Rubrobacter* type strains. It can be concluded that primers Rubro223f and Rubro454r are specific for the genus *Rubrobacter* and can be used to detect the presence and abundance of members of this genus in the Atacama Desert and other biomes.

**Electronic supplementary material:**

The online version of this article (10.1007/s10482-019-01314-3) contains supplementary material, which is available to authorized users.

## Introduction

The phylum *Actinobacteria* sensu Goodfellow (2012) contains several deeply branching lines of descent (Gao and Gupta 2012; Ludwig et al. 2012) including one composed of *Rubrobacter* species (Norman et al. 2017). *Rubrobacter*, the type and only genus in the family *Rubrobacteraceae* (Rainey et al. 1997; Zhi et al. 2009; Foesel et al. 2016) of the order *Rubrobacterales* (Rainey et al. 1997; Zhi et al. 2009; Foesel et al. 2016) of the class *Rubrobacteria* (Suzuki 2012a; Foesel et al. 2016) is loosely associated with taxa classified in the orders *Gaiellales* (Albuquerque et al. 2011; Foesel et al. 2016), *Solirubrobacterales* (Reddy and Garcia-Pichel 2009; Foesel et al. 2016) and *Thermoleophilales* (Reddy and Garcia-Pichel 2009; Foesel et al. 2016), all of which belong to the class *Thermoleophilia* (Suzuki and Whitman 2012; Foesel et al. 2016). Albuquerque et al. (2011) assigned two mesophilic strains isolated from a mineral aquifer in Portugal to the genus *Gaiella* as *Gaiella occulta*; the genus was assigned to the family *Gaiellaceae* of the order *Gaiellales*. Similarly, the order *Thermoleophilales* of the class *Thermoleophilia* (Suzuki and Whitman 2012; Foesel et al. 2016) includes the family *Thermoleophilaceae* (Stackebrandt 2005; Zhi et al. 2009; Foesel et al. 2016) and the genus *Thermoleophilum* (Zarilla and Perry 1984) which contains two thermophilic species, *Thermoleophilum album*, the type species (Zarilla and Perry 1984) and *Thermoleophilum minutum* (Zarilla and Perry 1986). In turn, the order *Solirubrobacterales* encompasses four families of mainly soil bacteria, the *Conexibacteraceae* (Stackebrandt 2005; Zhi et al. 2009; Foesel et al. 2016), *Parviterribacteraceae* (Foesel et al. 2016), *Patulibacteraceae* (Takahashi et al. 2006; Foesel et al. 2016) and *Solirubrobacteraceae* (Stackebrandt 2005; Zhi et al. 2009; Foesel et al. 2016) and associated species, including the type strains *Conexibacter woesei* (Monciardini et al. 2003), *Parviterribacter kavangonensis* (Foesel et al. 2016), *Patulibacter minatonensis* (Takahashi et al. 2006) and *Solirubrobacter pauli* (Singleton et al. 2003), respectively.

The genus *Rubrobacter* was proposed by Suzuki et al. (1988) to accommodate a γ-radiation resistant isolate from a hot spring in Japan and classified as *Arthrobacter radiotolerans* (Yoshinaka et al. 1973) prior to being renamed *Rubrobacter radiotolerans.* The genus description was emended by (Albuquerque et al. 2014). In general, *Rubrobacter* strains are obligately aerobic, Gram-stain positive, asporogenous, nonmotile actinobacteria which form irregular rods that occur singly, in pairs, tetrads and chains; the diamino-acid of the peptidoglycan is either l-lysine or *meso*-diaminopimelic acid; the predominant respiratory lipoquinone is MK-8, *iso*- and *anteiso*-fatty acids tend to prevail; their polar lipid patterns are complex, but usually include diphosphatidylglycerol and phosphatidylglycerol; and DNA G + C ratios fall within the range of 65–69 mol% (Suzuki 2012b).

In addition to the type species, the genus currently contains eight species with validly published names, *Rubrobacter aplysinae* isolated from the marine sponge *Aplysina aerophoba* (Kämpfer et al. 2014), *Rubrobacter bracarensis* from a deteriorated monument (Jurado et al. 2012; Albuquerque et al. 2014), *Rubrobacter calidifluminis* and *Rubrobacter naiadicus* from a fumarole heated stream in the Azores (Albuquerque et al. 2014), *Rubrobacter indicoceani* from a deep-sea sediment sample collected from the Indian Ocean (Chen et al. 2018), *Rubrobacter spartanus* from soil adjacent to the Kilauea volcanic caldera in Hawai (Norman et al. 2017), *Rubrobacter taiwanensis* from the Lu-Shan hot spring in Taiwan (Chen et al. 2004) and *Rubrobacter xylanophilus* from a thermally polluted effluent of a carpet factory in the United Kingdom (Carreto et al. 1996). The type strains of all but three of these species grow optimally at either 50 or 60 °C; *R. aplysinae* grows optimally at 25 °C and *R. bracarensis* and *R. indicoceani* at 28 °C (Jurado et al. 2012; Kämpfer et al. 2014; Chen et al. 2018). *R. radiotolerans*, *R. taiwanensis* and *R. xylanophilus* strains are remarkable for their resistance to high levels of γ-radiation (Yoshinaka et al. 1973; Ferreira et al. 1999; Chen et al. 2004), a property which may be conferred by stress genes, such as those involved in DNA repair homologous recombination, oxidative stress and compatible solute production (Egas et al. 2014).

Little is known about the ecology of *Rubrobacter* strains though they tend to be associated with extreme biomes, notably high temperature environments (Yoshinaka et al. 1973; Carreto et al. 1996; Ferreira et al. 1999; Chen et al. 2004; Albuquerque et al. 2014) while closely related strains have been isolated from Australian pasture soils (Janssen et al. 2002; Sait et al. 2002) and earthworm burrows (Furlong et al. 2002). In addition, culture-independent studies show that members of the genus *Rubrobacter* and closely related taxa are a feature of prokaryotic communities associated with rosy discoloured masonry and historic wall paintings (Schabereiter-Gurtner et al. 2001; Imperi et al. 2007), acid peat bog soil (Rheims et al. 1996), arid desert soils in Antarctica (de la Torre et al. 2003; Saul et al. 2005; Aislabie et al. 2006), Australia (Holmes et al. 2000; Janssen 2006) and Chile (Connon et al. 2007; Neilson et al. 2012; Crits-Christoph et al. 2013; DiRuggiero et al. 2013), heavy metal contaminated soils (Gremion et al. 2003; Moffett et al. 2003), as well as from Scottish grassland soils (McCaig et al. 1999) and earthworm burrows (Furlong et al. 2002). Holmes et al. (2000) designed an oligonucleotide probe, Rubro749, and used it to show that *Rubrobacter* and closely related taxa accounted for 2.6 and 10.2% of the bacterial flora of Australian Desert soils. These authors generated highly specific amplicons of *Rubrobacter* 16S rRNA genes from community DNA extracted from a desert environmental sample using the oligonucleotide probe in tandem with the universal primer 27f (Lane 1991). It is important to evaluate the effectiveness of such oligonucleotide primers given the addition of new 16S rRNA gene sequences to curated databases.

In the present study, a pair of oligonucleotide primers was generated and shown to distinguish the type strains of *Rubrobacter* species from representatives of the other aforementioned genera deeply rooted in the actinobacterial 16S rRNA gene tree. The primers were also used in pilot experiments designed to determine the presence of *Rubrobacter* clones in environmental DNA extracted from Atacama Desert soils.

## Materials and methods

### Source and maintenance of the strains

The source and key properties of the type strains of five *Rubrobacter* species and corresponding *Conexibacter*, *Patulibacter*, *Solirubrobacter* and *Thermoleophilum* strains are shown in Table 1, together with media used to cultivate them. All of the strains were maintained as slants on the appropriate agar media at room temperature and as 20% glycerol stocks at − 80 °C.

**Table 1:** Type strains of *Rubrobacter* species and those of related genera, their growth requirements and 16S rRNA gene sequence accession numbers

Strains	Source	Growth media	Optimal growth temperatures (°C)	16S rRNA gene sequence accession numbers	References
*Rubrobacter* *aplysinae*	DSM 27440^T^	DSMZ medium No. 514 + 1% starch	25	GU318365	Kämpfer et al. (2014)
*Rubrobacter bracarensis*	DSM 24908^T^	DSMZ medium No. 1350	28	EU512991	Jurado et al. (2012)
*Rubrobacter calidifluminis*	JCM 19154^T^	JCM medium No. 49	60	KF494338	Albuquerque et al. (2014)
*Rubrobacter naiadicus*	JCM 19155^T^	JCM medium No. 49	60	KF494339	Albuquerque et al. (2014)
***Rubrobacter radiotolerans***	JCM 2153^T^	JCM medium No. 49	37	X87134	Suzuki et al. (1988)
*Rubrobacter taiwanensis*	JCM 12932^T^	JCM medium No. 49	55	AF465803	Chen et al. (2004)
*Rubrobacter xylanophilus*	JCM 11954^T^	JCM medium No. 48	60	CP000386	Carreto et al. (1996)
*Conexibacter* *arvalis*	DSM 23288^T^	DSMZ medium No. 92	28	AB597950	Seki et al. (2012)
***Conexibacter*** ***woesei***	JCM 11494^T^	JCM medium No. 245	28	CP001854	Monciardini et al. (2003)
*Patulibacter americanus*	JCM 16550^T^	JCM medium No. 26	25	ATUD01000029	Reddy and Garcia-Pichel (2009)
*Patulibacter* *ginsengiterrae*	DSM 25990^T^	DSMZ medium No. 830	25	EU710748	Kim et al. (2012)
*Patulibacter* *medicamentivorans*	DSM 25962^T^	DSMZ medium No. 830	28	AGUD01000068	Almeida et al. (2013)
***Patulibacter minatonensis***	NCIMB 14347^T^	NCIMB medium No. 283	26	AB193261	Takahashi et al. (2006)
*Solirubrobacter* *ginsenosidimutans*	JCM 19086^T^	JCM medium No. 346	28	EU332825	An et al. (2011)
***Solirubrobacter*** ***pauli***	JCM 13025^T^	JCM medium No. 26	28	AY039806	Singleton et al. (2003)
*Solirubrobacter* *phytolaccae*	JCM 31078^T^	JCM medium No. 346	28	KF459924	Wei et al. (2014)
*Solirubrobacter* *soli*	JCM 14923^T^	JCM medium No. 346	28	AB245334	Kim et al. (2007)
*Solirubrobacter* *taibaiensis*	JCM 31079^T^	JCM medium No. 346	28	KF551107	Zhang et al. (2014)

Type strains of the type species of genera are given in bold

*DSMZ* Deutsche Sammlung von Mikroorganismen und Zellkulturen, *KCTC* Korean collection for type cultures, *JCM* Japan collection of microorganisms, *NCIMB* natural collection of industrial and marine bacteria

### Environmental samples

Four environmental samples were taken to represent different Atacama Desert habitats as shown in Table 2.

**Table 2:** Locations of environmental samples collected from diverse Atacama Desert habitats

Sampling site and code	Description of samples	Collection date	Latitude	Longitude	Altitude (m.a.s.l.)	Amplicons generated
Aguas Calientes (AC)	Halite encrusted soil from the edge of Salar de Atacama	04.11.16	23º08′79″S	67º25′29″W	4167	No
Quebrada Nacimiento (QN)	Sand near vegetation	04.12.17	23º37′06″S	67º50′56″W	3646	Yes
Salar de Tara (ST1)	Sand taken from under rock	06.11.16	23º02′97″S	67º18′87″W	4366	Yes
Valle de la Luna (VL)	Halite encrusted soil	04.11.16	22º55′08″S	68º19′20″W	2507	No

### Nucleotide sequences and bioinformatic analyses

16S rRNA gene sequences of the type strains of species classified in the genera shown in Table 1 were retrieved from GenBank (Benson et al. 2005) as were those of type strains of additional *Conexibacter*, *Patulibacter*, *Rubrobacter* and *Thermoleophilum* strains and of those of *Gaiella* and *Parviterribacter* species, as shown in Table S1. The corresponding sequence of *Escherichia coli* strain K-12 sub-strain MG1655 was accessed by its EcoGene number EG30084. Nucleotide alignments designed to identify conserved regions in *Rubrobacter* 16S rRNA genes were sought with the Clustal Omega (Sievers et al. 2011) webserver (https://www.ebi.ac.uk/Tools/msa/clustalo/) leaving the parameters in default mode. Nucleotide alignments were visualised in Jalview version 2 (Waterhouse et al. 2009); the position of nucleotides in the alignments followed *E. coli* 16S rRNA gene sequence numbering (Brosius et al. 1978; Yarza et al. 2014). In silico assessment of the specificity of the primers designed for the genus *Rubrobacter* was performed with the tool Probe Match available from the Ribosomal Database Project (RDP) server (https://rdp.cme.msu.edu/probematch/search.jsp) release 11 version 5, which has a repository of sequences of 16S rRNA genes of thousands of cultivable and non-cultivable bacteria (Cole et al. 2014). Additionally, the specificity of the primers was evaluated with Primer-BLAST software (https://www.ncbi.nlm.nih.gov/tools/primer-blast/) from the National Center for Biotechnology Information (NCBI) against a non-redundant database and default parameters (Ye et al. 2012).

### DNA extraction, PCR amplification and DNA fragment library construction

Genomic DNA was extracted from the strains shown in Table 1 using biomass grown for 10 days on the appropriate growth media at optimal temperatures. Biomass scraped from the surface of each of the agar plates, using sterile bacteriological loops, was washed twice in sterile water, resuspended in 0.5 ml of sterile distilled water and homogenised using micropestles. The extraction of genomic DNA was performed after Kieser et al. (2000). In turn, the extraction of environmental DNA from the environmental samples was achieved using a PowerSoil^®^ DNA Isolation Kit (MO BIO, Cat. No. 12888). Polymerase chain reactions (PCR) were carried out with Phusion High-Fidelity DNA polymerase (Thermo) using the GC buffer with 3% dimethyl sulfoxide following the manufacturer’s conditions. Ten ng of genomic DNA from the reference strains was used for the PCR amplifications under the following conditions: 98 °C for 2 min (initial denaturation); 30 cycles of 98 °C for 30 s (denaturation), 58 °C for 30 s (annealing) and 72 °C for 3 s (extension); 72 °C for 2 min (final extension); the annealing temperatures and the extension times were set after checking different values for these parameters. The resulting fragments were purified after electrophoresis in agarose gels using a GeneJET Gel Extraction Kit (Thermo Scientific, cat. No. K0691) and sent for sequencing to Macrogen Inc. (South Korea).

PCR amplicons obtained from the environmental DNA samples were purified from the agarose gels and cloned into plasmid pJET1.2/blunt using a CloneJET PCR Cloning Kit (Thermo Scientific, cat. No. K1231) following the manufacturer’s instructions. Transformations were carried out using *E. coli* DH5α as host and carbenicillin 50 μg/ml as the selective marker on Luria–Bertani agar (Difco). Positive clones were chosen by colony-PCR using Phusion High-Fidelity DNA polymerase and the forward primer pJET1.2 (5′-CGACTCACTATAGGGAGAGCGGC-3′) and the reverse primer pJET1.2 (5′-AAGAACATCGATTTTCCATGGCAG-3′) and grown overnight in 10 ml Luria–Bertani broth supplemented with 50 μg/ml carbenicilin for plasmid DNA extraction with a GeneJET™ Plasmid Miniprep Kit (Thermo Scientific, cat. No. K0502). This library of clones was sequenced using the pJET1.2 forward primer from Macrogen. The quality of the sequences were analysed using the Staden package (Staden et al. 2000) and the backbone vector sequence manually removed to obtain the final sequence fragments of the 16S rRNA genes amplified with the specific primers using the environmental DNA samples. Duplicated 16S rRNA gene sequences were identified using the ElimDupes tool from the HIV sequence database (https://www.hiv.lanl.gov/content/sequence/elimdupesv2/elimdupes.html).

### Phylogenetic analyses

The taxonomic affiliation of the 16S rRNA gene fragments obtained from the PCR runs with the designed primers were assessed in the EzBioCloud server (https://www.ezbiocloud.net) (Yoon et al. 2017) using the tool Identify. Phylogenetic trees were generated using the Genome-to-Genome Distance Calculator (GGDC; http://ggdc.dsmz.de/ggdc.php#) webserver (Meier-Kolthoff et al. 2013); visualised in FigTree version 1.4.2 (http://tree.bio.ed.ac.uk/software/figtree/).

## Results and discussion

### Design of genus-specific primers

**Fig. 1:**
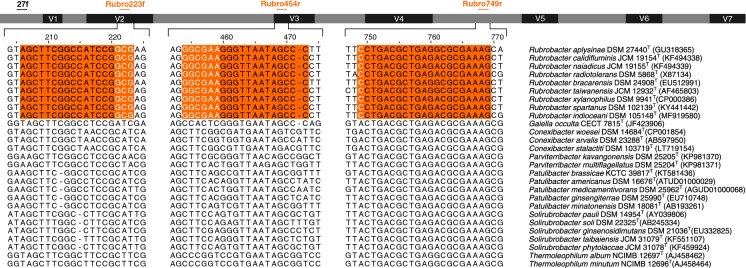
Conserved nucleotide regions of 16S rRNA genes of *Rubrobacter* type strains used to design the specific primers Rubro223f and Rubro454r. The bar represents the 16S rRNA gene sequence of *Escherichia coli*; black boxes indicate conserved regions and the grey ones variable regions (V) with corresponding numbers (Brosius et al. 1978; Yarza et al. 2014). Arrows above the bar represent the position of the primers within the 16S rRNA gene sequence. Nucleotide alignment for primers Rubro223f and Rubro454r and for primer Rubro749r (Holmes et al. 2000) are highlighted in orange boxes and nucleotides in white represent those unique to the genus *Rubrobacter* and hence absent in the type strains of species classified in the genera *Conexibacter*, *Gaiella*, *Parviterribacter*, *Patulibacter, Solirubrobacter* and *Thermoleophilum*. (Color figure online)

Conserved nucleotide signatures were sought in the 16S rRNA genes of the *Rubrobacter* type strains based on nucleotide alignments of 16S rRNA gene sequences and corresponding sequences of the type strains of species assigned to genera classified in the class *Thermoleophilia* (Fig. 1).

The DNA regions found to be specific to the 16S rRNA genes of the *Rubrobacter* strains were used as the starting point for primer design with unique 3′-ends for both forward and reverse primers. The first conserved region was 5′-GCG-3′ in positions 221–223 (*E. coli* numbering) and the second comprised 5′-GGCGAA-3′ in positions 454–460. These conserved regions were used to design a pair of primers for the amplification of a 267 nucleotide region within the range 223 to 454 of the 16S rRNA genes. The forward primer, Rubro223f (5′-AGCTTCGGCCATCCGGCG-3′) and the reverse primer, Rubro454r (5′-GGGCTATTAACCCTTCGCC-3′) consisted of 18 and 19 nucleotides, respectively.

The specificity of primers Rubro223f and Rubro454r was evaluated in silico using the RDP webserver (Cole et al. 2014) both individually and as a pair (Table 3). When tested, each primer was highly specific in detecting the 16S rRNA gene sequences of the *Rubrobacter* strains. In turn, when tested together, the specificity was above 98%. Additionally, electronic PCR (Ye et al. 2012) underlined the high specificity of the primers, as 99% of the hits corresponded to representatives of the genus *Rubrobacter* (Tables 1 and S2). Consequently, primers Rubro223f and Rubro454r were synthesised and used to validate in vitro PCR with genomic DNA extracted from the type strains shown in Table 1.

**Table 3:** Assessment of the specificity of primers Rubro223f and Rubro454r using the RDP database

Rubro223f		Rubro454r		Rubro223f/Rubro454r	
Actinobacteria	*Rubrobacter*	Actinobacteria	*Rubrobacter*	Actinobacteria	*Rubrobacter*
1049	1035 (98%)^a^	1058	1038 (98%)	717	717 (99%)^a^

^a^The hits are based on *Rubrobacter* strains deposited in the RDP database, apart from those of *R. spatanus* and *R. indicoceani* which were not available

### Validation of the primers with genomic DNA

**Fig. 2:**
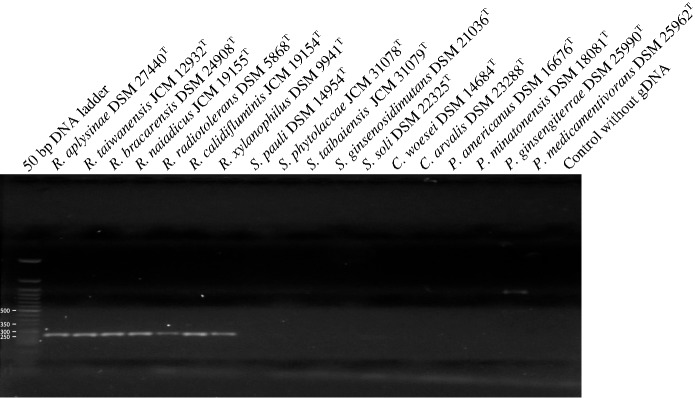
Verification of the specificity of primers Rubro223f and Rubro454r in PCR runs using genomic DNA extracted from *Rubrobacter* type strains and corresponding strains of the closely-related genera. Electrophoresis in 2% agarose gels shows PCR amplification of a region of 267 nt that was only found in the *Rubrobacter* strains

The primer set Rubro223f and Rubro454r was used to amplify the 267 nucleotide region of genomic DNA extracted from seven *Rubrobacter* type strains and from corresponding representatives of the closely related genera, as cited in Table 1. The primers enabled specific amplification of a region of the expected size from the genomes of the *Rubrobacter* strains (Fig. 2). Sequencing of these DNA fragments matched with corresponding sequences derived from the respective *Rubrobacter* strains. Consequently, it can be concluded that the primers specifically amplify a 267 nt fragment of the *Rubrobacter* type strains even though the region of genomic DNA amplified is short. These genus specific primers allow *Rubrobacter* strains to be distinguished from type strains of species assigned to genera classified in the class *Thermoleophilia* (Fig. S1).

### Use of the genus specific primers to detect Rubrobacter strains in selected Atacama Desert soils

Primers Rubro223f and Rubro454r were used to amplify community DNA extracted from environmental samples taken from four diverse habitats in the Atacama Desert; bands were obtained for the Salar de Tara (ST1) and Quebrada Nacimiento (QN) samples (Table 2). The purified amplicons were used to generate DNA libraries; 32 clones from sample QN and 37 from ST1 were selected and sequenced. The results from each of the sequences submitted to EzBioCloud (Yoon et al. 2017) showed that all of them corresponded to the genus specific fragment of the 16S rRNA gene characteristic of members of the genus *Rubrobacter*. Seventeen out of the 32 clones from sample QN represented unique clones; the corresponding figures from the ST1 sample were 16 unique sequences out of 37 clones. These results provide further evidence of the specificity of the primers. Future research is required to determine the adaptive mechanisms that have evolved in *Rubrobacter* strains to enable their survival in the harsh environmental conditions that prevail in the Atacama Desert.

### Phylogeny based on 16S rRNA gene fragments

**Fig. 3:**
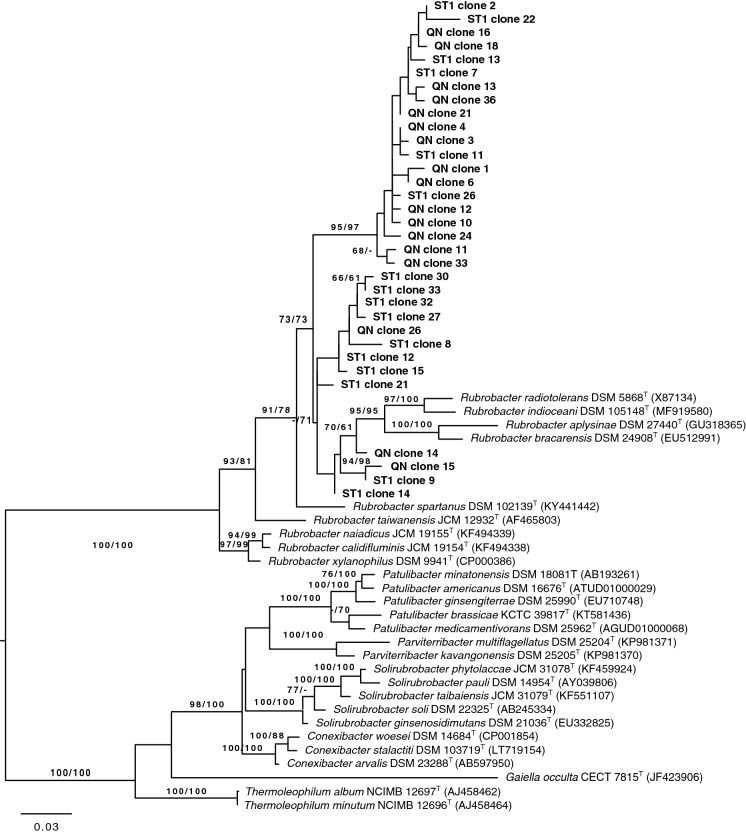
Maximum-likelihood phylogenetic tree generated using the GTR + CAT model and rooted by midpoint-rooting showing relationships between the 267 nt sequences amplified with primers Rubro223f and Rubro454r, using community DNA extracted from Salar de Tara (ST1) and Quebrada Nacimiento (QN) soils and the corresponding full 16S rRNA gene sequences of the type strains of representatives of the genera *Conexibacter*, *Gaiella*, *Parviterribacter*, *Patulibacter, Solirubrobacter* and *Thermoleophilum*. The branches of the tree are scaled in terms of the expected number of substitutions per site and the numbers above the branches are bootstrap support values greater than 60% for the ML (left) and MP (right) analyses

The 16S rRNA gene sequences of the clones generated from the PCR analyses of the community DNA were compared with corresponding sequences of the type strains of *Rubrobacter* species and those representing the genera *Conexibacter, Gaiella*, *Parviterribacter*, *Patulibacter*, *Solirubrobacter* and *Thermoleophilum*. It can be seen from Fig. 3 that all of the clones were recovered within the evolutionary radiation occupied by the genus *Rubrobacter*, an association supported by a 100% bootstrap value based on the maximum-likelihood and maximum-parsimony analyses. In contrast, the type strains of the other genera formed a second well defined clade. The type strains of *Conexibacter, Patulibacter* and *Thermoleophilum* also formed lineages supported by high bootstrap values that ranged from 66 to 100% (Fig. 3). It is also evident from the maximum-likelihood tree based on the 267 nt sequences of the type strains of all seven genera that the *Rubrobacter* strains fall into a distinct clade, as do those of the other six genera (Fig. S1). These well-defined taxa correspond to the classes *Rubrobacteria* (Suzuki 2012a) and *Thermoleophilia* (Albuquerque et al. 2011; Suzuki and Whitman 2012; Foesel et al. 2016).

Twenty out of the 33 clones (61%) were recovered in two lineages that were sharply separated from the *Rubrobacter* type strains (Fig. 3). The first taxon, which was supported by very high bootstrap values, encompassed 18 clones, 12 from QN and 6 from ST, while the second one contained two clones from the QN. In turn, clones QN14, QN15 and ST9 formed a well-supported phyletic line together with the type strains of *R. aplysinae, R. bracarensis*, *R. indicoceani* and *R. radiotolerans*, the type species of the genus; the final clone, ST15 was loosely associated with this lineage. These results provide further evidence that Atacama Desert soils are likely to be a rich source of novel *Rubrobacter* species (Connon et al. 2007; Neilson et al. 2012; Crits-Christoph et al. 2013; DiRuggiero et al. 2013).

It can be concluded that primers Rubro223f and Rubro454r are effective in distinguishing *Rubrobacter* strains from related actinobacterial genera classified in the class *Thermoleophilia* and in detecting the presence and abundance of 16S rRNA gene clones in Atacama Desert soils. They are also likely to be useful for detection of *Rubrobacter* strains in other natural habitats, as well as identifying colonies of *Rubrobacter* growing on isolation plates incubated at 28 and 50 °C (Carreto et al. 1996; Chen et al. 2004; Jurado et al. 2012), thereby providing a lead for bioprospecting, ecological and physiological studies on members of this poorly studied taxon. Such investigations are needed as it has been shown that culture based procedures grossly underestimate the extent of novel actinobacterial diversity within the Atacama Desert landscape (Idris et al. 2017; Bull et al. 2018) and in marine sediments (Stach et al. 2003).

It is also clear that new procedures are needed to cultivate members of *Rubrobacter* communities known to be present in natural bioemes, especially at a time when the ability to generate metagenomic data far outstrips the capacity to cultivate microorganisms as highlighted by Goodfellow et al. (2018), which also outlines promising new strategies for bringing previously uncultured bacteria into culture.

## Electronic supplementary material

Below is the link to the electronic supplementary material.

Supplementary material 1 (DOCX 262 kb)
