# Supplementary material for: New genus-specific primers for PCR identification of Rubrobacter strains
Source: Antonie Van Leeuwenhoek. 2019 Aug 12;112(12):1863–74. doi: 10.1007/s10482-019-01314-3 (PMC6834744; doi:10.1007/s10482-019-01314-3)
Supplement: Supplementary file 1 — Supplementary material 1 (DOCX 262 kb) [file 10482_2019_1314_MOESM1_ESM.docx]

**Supplementary Information**

**Table S1:** Source and 16S rRNA gene accession numbers of type strains of *Rubrobacter* and related taxa that were also used to devise primers Rubro223f and Rubro454r.

**Table S2**: Summary of the assessment of the specificity of the primers Rubro223f and Rubro454r in electronic PCR of NCBI and Probe Match of RDP.

**Fig. S1**: Maximum-likelihood phylogenetic tree obtained with the GTR+CAT model and rooted by midpoint-rooting showing relationships between representative of the genera *Rubrobacteria* with fragments of 267 nt of the 16S rRNA gene of *Rubrobacter* reference-strains and those of *Rubrobacteria* and *Thermoleophilia*, amplified with primers Rubro223f and Rubro454r. The branches are scaled in terms of the expected number of substitutions per site and the numbers above the branches are support values when larger than 60% from ML (left) and MP (right) bootstrapping. ST1: Salar de Tara; QN: Quebrada Nacimiento.

**Table S1**

| Strains | Source^*^ | Accession numbers | References |
| --- | --- | --- | --- |
| *Rubrobacter indicoceani* | DSM 105148^T^ | MF919580 | (Chen et al. 2018) |
| *Rubrobacter spartanus* | DSM 102139^T^ | KY441442 | (Norman et al. 2017) |
| *Conexibacter stalactiti* | DSM 103719^T^ | LT719154 | (Lee 2017) |
| ***Gaiella occulta*** | CECT 7815^T^ | JF423906 | (Albuquerque et al. 2011) |
| ***Parviterribacter kavangonensis*** | DSM 25205^T^ | KP981370 | (Foesel et al. 2016) |
| ***Parviterribacter multiflagellatus*** | **DSM 25204^T^** | KP981371 | (Foesel et al. 2016) |
| ***Patulibacter brassicae*** | KCTC 39817^T^ | KT581436 | (Jin et al. 2016) |
| ***Thermoleophilum album*** | NCIMB 12697^T^ | AJ458462 | (Zarilla and Perry 1984) |
| *Thermoleophilum minutum* | NCIMB 12696^T^ | AJ458464 | (Zarilla and Perry 1986) |

Type strains of the type species of the genus are given in bold.

^*^Key: CECT: Colección Española de Cultivos Tipo; DSMZ: Deutsche Sammlung von Mikroorganismen und Zellkulturen; KCTC: Korean Collection for Type Cultures; JCM: Japan Collection of Microorganisms and NCIMB: Natural Collection of Industrial and Marine Bacteria.

**Fig. S1**
